# Inherency of Form and Function in Animal Development and Evolution

**DOI:** 10.3389/fphys.2019.00702

**Published:** 2019-06-19

**Authors:** Stuart A. Newman

**Affiliations:** Department of Cell Biology and Anatomy, New York Medical College, Valhalla, NY, United States

**Keywords:** biogeneric, morphogenesis, pattern formation, cell differentiation, self-organization, phase transition, reaction-diffusion

## Abstract

I discuss recent work on the origins of morphology and cell-type diversification in Metazoa – collectively the animals – and propose a scenario for how these two properties became integrated, with the help of a third set of processes, cellular pattern formation, into the developmental programs seen in present-day metazoans. Inherent propensities to generate familiar forms and cell types, in essence a parts kit for the animals, are exhibited by present-day organisms and were likely more prominent in primitive ones. The structural motifs of animal bodies and organs, e.g., multilayered, hollow, elongated and segmented tissues, internal and external appendages, branched tubes, and modular endoskeletons, can be accounted for by the properties of mesoscale masses of metazoan cells. These material properties, in turn, resulted from the recruitment of “generic” physical forces and mechanisms – adhesion, contraction, polarity, chemical oscillation, diffusion – by toolkit molecules that were partly conserved from unicellular holozoan antecedents and partly novel, distributed in the different metazoan phyla in a fashion correlated with morphological complexity. The specialized functions of the terminally differentiated cell types in animals, e.g., contraction, excitability, barrier function, detoxification, excretion, were already present in ancestral unicellular organisms. These functions were implemented in metazoan differentiation in some cases using the same transcription factors as in single-celled ancestors, although controlled by regulatory mechanisms that were hybrids between earlier-evolved processes and regulatory innovations, such as enhancers. Cellular pattern formation, mediated by released morphogens interacting with biochemically responsive and excitable tissues, drew on inherent self-organizing processes in proto-metazoans to transform clusters of holozoan cells into animal embryos and organs.

## Introduction

Organization and functional integration are characteristics of multicellular organisms that distinguish them from mere associations of cells. Some organisms such as the social amoebae (e.g., *Dictyostelium discoideum*) (Bonner, 2009) and certain of the holozoans, a clade that includes both animal and nonanimal species (Sebé-Pedrós et al., 2017), exhibit both unicellular and multicellular life-cycle phases with a small range of differentiated cell types. But, it is in the animals or Metazoa, with up to hundreds of cell types constituting multifunctional, phenotypically plastic tissues, that organization and integration are most prominent. This review will focus on this group and the biological properties that distinguish it from its nearest relatives, the nonmetazoan holozoans.

There has been a wealth of approaches to the physiological relationships between, and evolutionary bases of, organization and integration in multicellular forms (see Arnellos et al., 2014 for a review). The main treatments have been within an evolutionary adaptationist perspective. These emphasize (1) the suppression of potential intercellular conflicts of genetically heterogenous cells leading to a “unicellular bottleneck” in development, (2) functional division of labor among cell types arrived at through cycles of natural selection for improved fitness, and (3) continuing evolution for integration among subsystems. Concepts that have been advanced to describe these processes include “alignment of fitness,” “export of fitness,” and “goal-directedness” (at the level of the organism as a whole), all of which, in most treatments, are proposed to be products of natural selection (Buss, 1987; Ruiz-Mirazo et al., 2000; Queller and Strassmann, 2009; Folse and Roughgarden, 2010). To this Arnellos et al. (2014) add the idea of “autonomy,” a concept related to function and goal-directedness, which has developed alongside the discourse on organization and integration (Ruiz-Mirazo et al., 2000; Ruiz-Mirazo and Moreno, 2004) and has been explored subsequently, in depth, in a monograph by Moreno and Mossio (2015). This property is seen as foundational to the possibility of Darwinian selection.

Following this line of thought, Keijzer and Arnellos (2017) have questioned whether animal-type autonomy can itself be accounted for by selectionist and adaptationist accounts and suggest that it might not. What is required is a type of organization, characteristic of animal bodies and organs, that distinguishes metazoans from other multicellular organisms like plants and fungi, and also from the unicellular populations that were their direct ancestors, some of which have extant forms with multicellular stages. In his foreword to the Moreno and Mossio volume, Cliff Hooker problematizes the kind of organization that is found in its most intensive form in animals:

Organisation is not the same as order; pure crystals are highly ordered, so uniform they cannot show any organisation. Neither can gases, because they are too random to be organized. Organisation lies between the crystal and gas extremes but we don’t have a good theory that tells us exactly where and why. (Moreno and Mossio, 2015, p. vii)

I suggest that recent work on the mesoscale physics (i.e., processes at the 10^−3^–10^−2^ m scale) of cell collectives and on the products of the genetic toolkit that mediate these cell associations in animal tissues permit us to formulate a theory of the organizational properties of metazoan organisms. Specifically, I draw on earlier work on “dynamical patterning modules” (Newman and Bhat, 2008, 2009), developmental units in which the products of certain conserved genes mobilize physical forces and processes at the multicellular scale, and on “biogeneric materials” (Newman, 2016a). The latter are condensed forms of matter (in Hooker’s formulation, “lying between the crystal and gas extremes”) that, while arising from collections of living cells, exhibit predictable morphological motifs due to their having properties in common with nonliving materials such as liquids and elastic sheets. These concepts can help us understand how and why certain developmental forms and patterns of animal bodies and organs (multilayering, lumens, segmentation, appendages) have appeared recurrently throughout evolution.

In addition to their morphological characters, animal bodies exhibit coordinated and integrated functioning of distinct cell types. These alternative states of cellular activity, which depend on, but are not entirely reducible to differential gene expression, are partly conserved across the animal phyla, and the means of their progressive emergence from unicellular ancestors of the metazoans are only now becoming known (Brunet and King, 2017; Sebé-Pedrós et al., 2018). While the range and stereotypical character of the cell types of animals are not inherent to multicellular matter in the same way that the morphological motifs of these organisms are, their functions are intrinsic to the biology of their ancestral cells. The partitioning of these functions among up to several hundred different cell types depended on certain innovations in gene transcriptional regulation. The relations between the mechanisms of gene expression employed in the animals’ immediate unicellular ancestors and those regulatory mechanisms that distinguish them from other kinds of organisms are key to their ability to allocate inherent functions to specialized cells and will therefore be described in detail.

Beyond the inherency of tissue morphology and cell function described above, the development and evolution of complex bodies and organs involves a third category of processes that depends on, but is not a mere combination of, these two: the reliable spatial arrangement of different cell types within three-dimensional tissues (Salazar-Ciudad et al., 2003). These processes, collectively termed cellular *pattern formation*, are also inherent to animal systems, but in a way still different from morphogenesis and differentiation. They arise from the fact that biogeneric materials, having subunits that are living cells, are dynamically more complex than their nonliving counterparts. In multicellular systems, short- and long-range cell-cell communication *via* contact, chemical and mechanical signaling or electrical coupling, along with positive and negative feedback effects, produce lateral inhibitory effects, oscillations, synchrony, and repeated structures. Together, the animal-specific set of morphogenetic, differentiative, and patterning inherencies appear to be the organizational preconditions of subsequent metazoan evolution.

For each of the processes I discuss I will describe the main genes and molecular mechanisms involved in distinguishing animals in general from their inferred unicellular ancestors and some of the major organizational differences within Metazoa from one another. This has led to the identification of biological properties dependent on (1) novel genes or regulatory motifs coincident with emergence of Metazoa, (2) novel genes acquired after metazoan origins, and (3) ancestral genes repurposed to novel functions when they came to operate on the multicellular scale (Table 1).

**Table 1:** Novel inherent properties in animal development and evolution.

Property	Gene or molecular motif	Character
1. Properties dependent on novel genes or regulatory motifs coincident with emergence of Metazoa Liquid-tissue state Regulated cell polarity Capacity to exaggerate intrinsic cell functions Morphogen gradients	Classical cadherins Wnt Enhancers; PcGI proteins Hedgehog, BMPs	Multicellularity; layering Lumens; elongated tissues Differentiation Simple cell patterns
2. Properties dependent on novel genes acquired after metazoan origins Liquid-crystalline-tissue state Wettable substrata (basal lamina) Lateral inhibition; oscillation of gene expression Multiple alternative cell types	Vang/Stbm Peroxidasin Notch, Hes1 MyoD, PPARγ, SMAD	Tissue elongation Appendages, glands Complex cell patterns Complex tissues, organs
3. Properties dependent on ancestral genes repurposed into DPMs in the multicellular context Cell-cell cohesion in liquid tissues Apicobasal cell polarization Nonliquid cellular assemblages *via* matrices Cell-cell electrical coupling	Grainyhead β-catenin Collagen IV Voltage-gated channels	E-M Transformation Epithelia and lumens Mesenchymal tissues Bioelectrical integration

Each list is nonexhaustive but contains the most important examples of its respective category.

If, as I suggest, inherency of form, function, and pattern make key features of animal body plans [and, as we propose elsewhere, those of plants (Benítez et al., 2018)] highly constrained and readily achieved, the role of natural selection for incrementally improved fitness becomes less decisive as the driving force of organismal diversification and complexification than generally believed. In the concluding section, I will discuss the relationship between these evolutionary perspectives.

## Inherent Forms in Animal Origination and Evolution

In this section, I discuss the structural motifs that would inescapably characterize the metazoans once they emerged from an ancestral population of unicellular organisms. These characters were due to animal tissues being constituted as a new form of *mesoscale matter* by the innovation of molecular links between cells that also harnessed the cells’ motile activity. The physics of these living materials generated a specific array of morphological motifs with a constrained range of geometric and topological relationships among them. Later, with the appearance of additional novel genes (some of them phylogenetically unprecedented), new physical forces and constraints were mobilized in some of these multicellular entities, leading to successively more complex forms. The morphological properties described, which can be identified with phylotypic bodies and organs, were generally predictable properties of these materials and therefore inherent to animal tissues.

### The Liquid-Tissue State: The Defining Character of Metazoa

The animals or metazoans arose around 700 million years ago within an evolutionary lineage of eukaryotic cells – the holozoans – which were also ancestral to present-day unicellular choanoflagellates (reviewed in Newman, 2016b). The animals are multicellular, and in all metazoan phyla cell-cell attachment is mediated by members of the cadherin family of cell adhesion molecules (CAMs). Organisms of all these phyla express cadherins with a cytoplasmic domain that links to the actin cytoskeleton and permits cells to remain tightly bound to their neighbors, while they move past one another (reviewed in Newman, 2016a). With one exception [the ctenophores – “comb jellies” – a group that is an outlier of the metazoans in several respects (Lanna, 2015; King and Rokas, 2017; Whelan et al., 2017)], cell-cell attachment during development is mediated by the so-called “classical” cadherins (reviewed in Newman, 2016). These proteins are present in the morphologically simplest, and earliest diverging, animals, the sponges (Porifera) and the single extant species of Placozoa, *Trichoplax adhaerens*, the so-called *basal metazoans*. The cytoplasmic domain of the classical cadherins is not found in any nonmetazoan holozoans and has no counterpart in any other sequenced organisms. [The cadherins of ctenophores have a different cytoplasmic domain (Belahbib et al., 2018).] Such unprecedented coding sequences have been termed “*de novo*” or “orphan” genes (Bornberg-Bauer et al., 2015; Schmitz et al., 2018).

Materials with subunits that cohere but can nonetheless perambulate in a random fashion are, by definition, liquids. They exhibit surface tension and the propensity (like oil and water, for example) to phase-separate from other such materials with different cohesive properties. The liquid properties of metazoan cell clusters are based on biological functions rather than purely physical attributes. For example, in nonliving liquids, cohesion of molecular subunits is mediated by electrical forces, and the random movement of those subunits is due to Brownian motion driven by thermal energy (March and Tosi, 2002). In animal tissues the subunits are cells, whose cohesion is mediated by classical cadherins and whose random movement results from surface protrusive activity and amoeboid motion driven by cytoskeletal mechanics. Nevertheless, the physical laws that describe these two categories of liquid are sufficiently similar, so that many properties of the biological materials are predictably “generic” (Newman and Comper, 1990; Forgacs and Newman, 2005). Liquid tissues and other cell collectives with biologically based generic physical properties are thus termed *biogeneric* (Newman, 2016a).

The “liquid-tissue” state enabled by metazoan cadherins was a primitive defining condition of animal life. Liquid tissues have several emergent features that appeared independently of natural selection in the transition between ancestral colonial holozoans and ancestral metazoans. Liquid droplets, for thermodynamic reasons, assume the geometry with smallest surface-to-volume ratio, a sphere. Embryos and newly formed organ primordia are therefore spherical by default (Steinberg and Poole, 1982). In liquids that contain two different kinds of subunits (molecular species, in most physical examples), one of which has greater affinity for its own type than the other (due, in liquid-tissues, to different amounts or types of cadherins on cell surfaces), there is a separation of phases. The interface between the phases can be flat or curved, depending on the balance of self- and nonself-affinities, and in extreme cases, one liquid phase will completely engulf the other. Such configurations are common in cell mixing and sorting experiments (Forgacs and Newman, 2005). Gastrulation, the organization of a coherent mass of cells into closely apposed but nonmixing layers, which is the first step in body plan formation during development, is directly attributable to phase separation in some animal systems (Krieg et al., 2008). In other organisms, where the connection to phase separation is not as direct, more elaborate mechanisms of gastrulation may have built on morphological templates arising from this inherent tissue behavior early in their evolution (Newman, 2016a).

Phase separation of cell populations in a liquid tissue mass illustrates the concept of dynamical patterning modules (DPMs) mentioned above (Newman and Bhat, 2008; Newman et al., 2009). In this example, cells with different homotypic and heterotypic adhesive strengths undergo spontaneous spatial redistribution to a predictable pattern by mobilizing, in multicellular clusters, a physical force (cell-cell adhesion) that is irrelevant to individual cells. The molecules that mobilize the physical effect are classical cadherins, which arose concomitantly with the metazoans. Other such examples of DPMs co-originating with Metazoa will be described in what follows. But in still other cases, proteins that evolved in unicellular ancestors took on novel DPM roles in the multicellular context, or novel gene products acquired later in animal evolution took on developmental functions by harnessing new physical processes in the form of DPMs.

In addition to having cytoskeletally linked classical cadherins, the metazoans are distinguished from all other life forms by the protein Wnt, which is similarly specified by an orphan gene unique to this group of organisms (reviewed in Newman and Bhat, 2009)^1^. Wnt induces reorganization of the cytoskeleton, acting *via* surface receptors (e.g., Frizzled, LRP5/6), β-catenin [itself unique to Metazoa among its close unicellular relatives, but with a homolog in the more distantly related *D. discoideum* (Miller et al., 2013; Gul et al., 2017)], and the PAR complex of adapters and protein kinases (Lang and Munro, 2017). The PAR complex predated the metazoans and serves to make the surfaces of cells nonuniform by mediating the insertion of different sets of integral membrane proteins on different portions (e.g., apical, basolateral) of the plasma membrane. In a manner that is physically analogous to polar molecules spontaneously organizing into micelles in water, liquid tissues containing apicobasally (A/B) polarized cells spontaneously form lumens and interior spaces (Forgacs and Newman, 2005).

### Planar Liquid-Crystalline Tissues and the Eumetazoans

To move beyond the simple forms of sponges and placozoans, two additional features were required, *planar cell polarization* (PCP) and the capacity to form a *basal lamina*. These effects, in turn, required several molecular innovations, using genes not present in the basal metazoans. One of these effects is a “noncanonical” Wnt-activated signaling pathway that reorganizes the cytoskeleton *via* the PAR complex but does so (in contrast to the canonical pathway described above) in a β-catenin-independent fashion. This pathway is facilitated by the inhibitory effect of the β-catenin antagonist Vang/Stbm, still another orphan gene, in this case unique to the *eumetazoans*, animals that develop from two or three distinct embryonic layers (diploblasts and triploblasts). The PCP pathway may have arisen in the common multicellular ancestor of the metazoans, but key components were lost in most sponge groups and placozoans, and it is incompletely present in ctenophores (Schenkelaars et al., 2016).

Unlike the canonical Wnt pathway, the PCP pathway causes cells to be polarized in their shapes or spatial orientation rather than over their surfaces. In a multicellular context, cells that elongate due to PCP align and intercalate, which causes the tissue mass to narrow in the direction of intercalation and elongate orthogonally to it. This tissue reorganization, termed *convergent extension* (reviewed in Forgacs and Newman, 2005), is the biogeneric counterpart of the deviation, in liquid crystals, from the spherical default shape of a liquid droplet due to alignment of polymers or anisotropic nanoparticles (reviewed in Newman, 2016).

The evolutionary appearance of these “liquid-crystalline tissues” was accompanied by the capacity to form *epithelia*, planar tissues whose cells reside on a stiff extracellular layer, the *basal lamina*^2^. Direct cell-cell attachment in such tissues continues to be mediated by cadherins, as in the *epithelioid* mode of tissue organization that characterizes some nonplanar tissues in all metazoans. But the basal lamina constitutes a substratum upon which the liquid-tissue can adhere and spread, a physical process referred to as “wetting” (Jensen et al., 2015). Depending on the relative degree of affinity between the basal regions of the apicobasally polarized cells that produce the basal lamina and the substratum itself, and their lateral affinity to one another, the epithelium will be columnar, cuboidal, or squamous.

Basal laminae are absent in the placozoan and most sponge species but are present in all eumetazoans (reviewed in Newman, 2016). The basal lamina is predominantly composed of the extracellular matrix (ECM) protein type IV collagen, which is produced by all metazoans, but only in the more morphologically complex, eumetazoans and the homoscleromorph sponges are its molecular subunits chemically crosslinked into a basal lamina (Fidler et al., 2017). This crosslinking is catalyzed by the enzyme peroxidasin, which is specified by an orphan gene unique to this group of organisms (Fidler et al., 2014)^3^. The capacity to form epithelia, in coordination with ability of the main body mass and subsidiary buds to undergo convergent extension, permitted diploblastic, and eventually triploblastic, eumetazoans to generate elongated bodies, appendages, tentacles, and tissue ridges, folds, and clefts. These morphological motifs were not only enabled by these new tissue organizational properties, but they were also made readily achievable.

### Epithelial-Mesenchymal Transformation, Mesenchymal Condensation, and Triploblasty

With the evolutionary emergence of a third tissue layer, which came to be sandwiched between the two epithelial germ layers in one or more diploblastic ancestors, the *triploblasts*, the most ecologically successful of the metazoan groups, came into existence (reviewed in Newman, 2016). The middle layer – mesoderm – enabled generation a new array of morphological motifs, which were correspondingly inherent to these new organisms. These intrinsic organizational propensities led to triploblasts having more complex body plans than diploblasts, and, in contrast to the latter, geometrically complex organs containing multiple cell types. An important reason for this was that the surface and enteric epithelia in triploblasts could continue to serve (as in diploblasts) as a boundary between the organism and its environment, while the mesodermal layer, having no analogous protective role, could disaggregate into a new physical state consisting of cells dispersed in a matrix, a *mesenchyme*. Mesenchymal tissues, on their own, or in interaction with an adjacent epithelium, could form structures that were impossible in simpler animals, but predictable, based on the additional physical effects to which they were susceptible (reviewed in Newman, 2016b).

The morphologically simplest triploblasts develop with a single body cavity, the digestive tube. Such organisms, including flatworms like planaria, have ovaries and testes, and clusters of neurons, termed ganglia. Most triploblasts, however, such as arthropods, mollusks, and chordates, have a second body cavity, the coelom, within which digestive tube is suspended. In these coelomate triploblast lineages, organ complexity took a dramatic leap. This is because instead of their being just two epithelial-mesenchymal interfaces, as in acoelomates, there were now four. The interaction of the epithelium of the body surface (the ectoderm) with its underlying mesenchyme produced, in various species, bristles, hairs, feathers, teeth, and limbs, while the interaction of body lining epithelium (the endoderm) with its overlying mesenchyme produces intrinsic and extrinsic elaborations of the digestive tube, respectively, villi and crypts, and the liver and pancreas. The blood vessels and heart, lungs, and urogenital organs, as well as various glands are generated by invagination and evagination, folding and branching, and thickening and thinning of composite epithelial-mesenchymal layers in other regions of the developing embryo (reviewed in Newman, 2016a).

Similar to the rise of the animals as a whole, and in their turn, the eumetazoan subgroup, the appearance of the triploblasts was accompanied and facilitated by novel gene products. These included structural ECM molecules such as fibronectin and tenascin (Hynes, 2012), glycans bearing unusual sugar moieties, and enzymes such as Has2 [which may have had a viral origin (Blackburn et al., 2018)] that generate ECM polysaccharides such as hyaluronan, which induce cell motility (Spicer and Tien, 2004). These molecules led to the ingression, typically accompanied by disaggregation of an epithelial layer (*epithelial-mesenchymal transformation*; EMT), in one or more diploblastic ancestors. Because of the heterogeneity of the mesoderm-inducing components, it is possible that triploblasts might not be monophyletic (Burton, 2008).

Mesenchymes and their mature counterparts, connective tissues, lack direct integration of cell-cell attachment with cytoskeletally driven motility and are therefore not liquid tissues by the definition above. They are nonetheless biogeneric materials, variously viscous, viscoelastic, or solid, depending on the composition of their ECMs or (as described below) the state of mechanical stress of the contained cells. As with liquid- and liquid-crystalline tissues, mesenchymal tissues have characteristic inherent forms. One prominent example is *cell condensation*, wherein groups of cells embedded in ECM are drawn closer to each other (a structural motif unavailable in epithelioid tissues), forming transient clusters with epithelial-type contacts. Condensations serve as the primordia of hairs, teeth, and feathers, as well as the bones of vertebrate fins and limbs (reviewed in Forgacs and Newman, 2005).

Cnidarians, which are diploblasts, can form solid-phase exoskeletons consisting of calcium-based mineral or the polysaccharide chitin. In later diverging triploblastic forms such as bivalve mollusks and arthropods, exoskeletons continue to be produced by epithelia. But the presence of mesoderm enables some triploblasts, such as echinoderms and chordates to also produce endoskeletons (reviewed in Brown, 2011). These are typically based on patterned arrays of mesenchymal condensations (Hall and Miyake, 2000) and become solid by the production of collagenous ECMs, containing a high density of proteoglycans in cartilage, or hydroxyapatite mineral in bone and the dentin of teeth. (Tooth enamel is a mineralized exoskeletal tissue.)^4^

Mesenchymal tissues can also undergo a different rigidifying transformation termed “fluid-to-solid jamming” (Mongera et al., 2018). This is a phase transition between the liquid-tissue state and a solid state [analogous to glass transitions in supercooled fluids (Sadati et al., 2014)], in which persistent cytoskeletally mediated stresses at a supracellular scale solidify the tissue. Jamming is employed as a mechanism (alternative to convergent extension) of elongation of the primary body axis in zebrafish embryos. It is impaired by knockdown of a cadherin, suggesting that it depends on a shift from mesenchymal ECM-based tissue cohesion to an epithelioid mode of cohesion, like mesenchymal condensation. The nominally “solid” phase after jamming therefore exhibits some liquid-tissue properties. Specifically, cell-scale stresses fluctuate rapidly, enabling cell rearrangements, thus effectively melting the tissue at the growing end and permitting longer-term supracellular-scale stresses to guide morphogenetic flows in fluid-like tissue regions.

Metazoan tissues thus have inherent morphogenetic propensities due to their liquid, liquid-crystalline, planar, and solidifying nature. These comprise mutlilayering and lumen formation (which is seen in all groups, even the most basally diverging ones), body elongation and appendage formation (which are seen in all eumetazoans), and organ formation (found in all triploblasts). The bodies of triploblasts can also become segmented by the deployment of oscillatory processes intrinsic to cells and tissues (see below, “Pattern Formation: Inherency in the Integration of Form and Function”). The structural motifs of organs, tissue multilayers, folds, ridges and projections, straight and branched tubes, segments and lobes resemble those that produce the phyletic body plans and employ the same conserved set of developmental toolkit genes. Since the relevant biogeneric properties, the genes that mobilize them, and the inherent forms they facilitate, arose very early in the history of the metazoans, gradualist natural selection did not bear the exclusive burden of producing the major structural features of animal bodies and organs.

Morphological motifs are not cell types, however, and it is through the functional specializations of their cells that the physiology of animal life is realized. I describe next how ancestral and derived inherency rather than classical gradualist trial-and-error scenarios plausibly accounted as well for the evolution of cell differentiation in metazoans.

## Inherent Cell Functions in Animal Origination and Evolution

Just as the structural motifs of animal bodies (e.g., layers, lumens, segments) are inherent to the materials that constitute them, so are the functional capabilities (e.g., contractility, excitability, detoxification) that came to be reflected in differentiated cell types, tissues, and organs. The reasons are not the same, however. Notwithstanding speculations based on the dynamical multistability of gene regulatory networks (GRNs; Kauffman, 1969; Furusawa and Kaneko, 2002), the full range of different cell types compatible with an organism’s genome does not seem to be the global state-space attractors of any unitary physical system. Here I do not mean to suggest that the nonlinear dynamics of GRNs and transitions between adjacent attractors of such systems are not suitable accounts of progression along developmental lineages, e.g., Huang et al. (2007). Such steps take place in functionally delimited domains of the full gene expression state space, with modest combinatorial differences in transcriptional determinants. There are persuasive examples of such phenomena (Mojtahedi et al., 2016; Corson and Siggia, 2017; Verd et al., 2017). However, the differential equation or Boolean representations of GRNs usually employed in the global models have long been criticized on conceptual grounds (Rosen, 1991) and have been further called into question by new findings on transcription factor disorder and condensed state chemistry reviewed in this section (see Niklas et al., 2015).

Instead, I suggest that the inherency of the specialized functions of multicellular organisms, such as animals and plants, resides in the fact that they are exaggerated counterparts of life-sustaining activities of their ancestral unicellular forms. Cellular functions in general have a history that goes back to the transition between the nonliving and living state (Arnellos and Moreno, 2012) but are not entirely shared in all clades. Some differentiated cell types amplify what are universal cell functions, but others draw on more directly ancestral physiology.

In this section, I show how cell types likely arose through the recruitment and partitioning of ready-made ancestral cellular functions by a highly plastic metazoan-specific gene regulatory apparatus. Although based on the “write and read” histone modification language that had evolved in the earliest eukaryotes (Prohaska et al., 2010), this apparatus is unlike that of any extant unicellular relatives. It appeared coincidentally with the metazoans, incorporating elements found in present-day holozoan and more distantly related fungi, as well as some unique elements not found in any other known life-forms. The new gene regulatory mode came to operate in a multicellular context that (as I will show below in relation to the role of the ancestral grainyhead transcription factor) was brought about by some of the same effects. Once metazoan lineage-defining transcription factors appeared (an evolutionary step that is currently enigmatic), partitioning of ancestral functions into differentiated cell types followed in a straightforward and potentially rapid fashion.

To reiterate, although metazoan-type mechanisms of cell differentiation were not inherent in unicellular progenitors, most cellular functions that came to be specialized in differentiated cell types were. Furthermore, once the enhancer-based, developmentally hierarchical means of exaggerating gene activity were in place, the evolution of developmental lineages of initially related, but progressively divergent, cell types was readily achieved.

### Enhancers, Silencing Complexes, and the Gene Regulatory Ground State of the Metazoans

While the genes of all categories of organisms are regulated by nearby upstream cis-acting promoters, the additional effects of enhancer sequences, which can be located upstream or downstream from their targets, or in introns, are, with the apparent exception of Placozoa, hallmarks of metazoan gene control. Enhancers are novelties of Metazoa or its direct antecedents: they are absent in extant unicellular holozoans (Sebé-Pedrós et al., 2016). There are more enhancers than regulated genes [as many as 50,000 in mammalian genomes, for example (Heinz et al., 2015)], and while their TF-binding motifs are similar to those of promoters (Arenas-Mena, 2017), they function differently. Enhancers mediate the high levels of expression of the characteristic genes of terminally differentiated cells, as well as the precise spatiotemporal generation of cell and tissue types during development due to their responsiveness to patterning cues (Lenhard et al., 2012; Zabidi and Stark, 2016) (see section “Pattern Formation: Inherency in the Integration of Form and Function”).

Enhancers show not only evidence of conservation but also divergent usage over evolution and between species (Chen et al., 2018). They are employed in sponges and all eumetazoans, with an indication of their presence in ctenophores, although not the placozoan *T. adhaerens*, which appears to rely solely on promoters to generate its few cell types (Sebé-Pedrós et al., 2018). Enhancers regulating stage- and cell type-specific “developmental” and “terminal differentiation” genes are often located at significant linear distances from the respective promoters and interact with a partly different set of TFs from the more proximal enhancers regulating ubiquitously expressed “housekeeping” genes (Zabidi et al., 2015).

The origin of enhancers may reside in the duality of promoter functional architecture in ancestral cells. Unicellular holozoans have inducible promoters that respond to external cues as well as promoters that regulate constitutive genes, with the latter often exhibiting distal cooperative interactions. Arenas-Mena (2017) has proposed a scenario by which inducible promoters were converted in few steps to a distally acting enhancer mode of developmental gene regulation in metazoans.

In metazoans, chromatin loops containing cell-type regulated DNA sequences enter or exit distinct liquid-phase proteinaceous condensates along with other loops containing enhancers and a large multi-subunit obligatory regulatory complex called Mediator, forming “topologically associating domains” (TADs) within the interphase nucleus (Galupa and Heard, 2017; Furlong and Levine, 2018; Plys and Kingston, 2018). Thousands of enhancers can be recruited to a given gene by lineage-determining transcription factors (LDTFs) (Heinz and Glass, 2012; Link et al., 2015) such as the muscle “master regulator” MyoD (Blum and Dynlacht, 2013). This occurs in a highly cooperative and synergistic fashion, with the number of enhancers recruited being responsive to developmental cues external to the differentiating cells.

Transcription in animal cells is initiated by the action of the master coactivators p300 and CBP (Chan and La Thangue, 2001). These highly homologous multidomain proteins contain histone acetyltransferase domains that relax the structure of nucleosomes at promoters and enhancers of all or most transcribed genes, enabling the recruitment of RNA polymerase II and TFs to those sites. Mediator, p300/CBP, and TFs all contain intrinsically disordered regions (IDRs) that assume different 3D structures under different conditions (Liu et al., 2006; Minezaki et al., 2006). The IDRs are required for the formation of nuclear TADs and are the basis for p300/CBP’s role as an interaction hub with a multitude of TFs and coregulators [most prominently nuclear receptors (NRs)] that mediate tissue responses to developmental and physiological cues (Dyson and Wright, 2016). Expression of a specific gene is caused by p300/CBP binding to transactivating protein domains (also abbreviated as TADs) of the TFs and NRs targeting the gene’s promoter, followed (based on evidence from some “super enhancers”) by induction by TF TADs of phase separation of Mediator into activating droplets (Boija et al., 2018).

Extant unicellular holozoans such as *Capsaspora owczarzaki* contain p300/CBP, and their promoter nucleosomes exhibit p300/CBP-specific histone acetylation marks. This indicates that the gene regulatory role of p300/CBP is phylogenetically older than that of enhancers (Grau-Bové et al., 2017). These coregulators contain bromodomains, which in metazoans promote enhancer-dependent expression of developmental and cell-type specific genes (Wei et al., 2008). Metazoans also express the bromodomain-containing protein Brd4, which co-localizes with LDTFs on active enhancers, where it recruits Mediator and RNA polymerase II (Lee et al., 2017).

Suppressing some genes can be as important as activating others in shaping cell type identity. In metazoan development and terminal cell differentiation, this is accomplished by “silencing” complexes such as the Polycomb-group (PcG) proteins (Khan et al., 2015) and other lysine methyltransferases such as SUV39H (Jih et al., 2017). These inhibit gene function by methylating histone 3 in the nucleosomes on both promoters and coding regions of genes that are inactivated at developmental stages and in terminally differentiated cell types, but not in the nucleosomes of housekeeping genes (El-Sharnouby et al., 2017). Silencing mechanisms based on PcGII proteins evolved in a common ancestor of holozoans and fungi and were retained in yeast and presumably the direct holozoan progenitors of Metazoa (Kingston and Tamkun, 2014; Steffen and Ringrose, 2014), though they were lost in the unicellular holozoan *C. owczarzaki* (Grau-Bové et al., 2017). Silenced sequences are converted into stably inactive heterochromatin by PcGI proteins, which are metazoan innovations (Grossniklaus and Paro, 2014; Steffen and Ringrose, 2014). Like transcription hubs, silencing complexes are phase separated into biomolecular condensates (Tatavosian et al., 2019), and their association with the latter appears to be regulated by short-lived noncoding RNAs (eRNAs) transcribed from enhancers (Chan et al., 2018).

To summarize, gene activation mechanisms based on p300/CBP activating and PcGII and SUV-type gene silencing mechanisms were conveyed to metazoans from ancestral unicellular holozoans, while enhancers and PcGI gene heterochromatin sequestration mechanisms accompanied the origination of these organisms. Understanding the emergence of cell differentiation, then, requires focusing on an inferred population of holozoan organisms, which, unlike other members of this group, contained enhancers, which are promiscuous and condition dependent in their gene activating activities, and silencing complexes, which since they depend on enhancers, are also subject to microenvironmental input. These organisms also contained β-catenin, a component integral to metazoan cell adhesion (see section “Inherent Forms in Animal Origination and Evolution”) that is also a key element of externally regulated gene expression. This was the biological “ground state” of metazoan evolution.

### Grainyhead and the Origin and Stabilization of Metazoan Multicellularity

It is unknown whether multicellularity preceded or followed the appearance of the described metazoan-specific modes of gene regulation in the ancestors of animals. But for a single organism’s cells to diversify into functionally specialized types, multicellular clusters must already exist. At the level of gene regulation, this requires factors that promote cell-cell adhesion and inhibit cell-cell detachment. The metazoan innovations of the classical cadherin cell attachment molecules and their associated cytoskeletal adaptors have been discussed above. This is one side of the multicellularity dyad. Cell division is commonly followed by cell separation, however, and this function must be suppressed for organismal identity to be determinate.

The grainyhead (Grh) family of TFs has a deep evolutionary history in the opisthokonts, i.e., the fungi and holozoans, including all metazoans, where they are intimately involved in epithelial organ development and epithelial barrier repair after tissue damage (Wang and Samakovlis, 2012). In the fungus Neurospora, the TF GRHL has a DNA-binding specificity close to that of animal grainyhead proteins (Pare et al., 2012). Moreover, Neurospora grhl mutants [allelic with conidial separation-2 (csp-2) mutants] are defective in spore dispersal. A conserved role for Grh-like TFs in the development and remodeling of cell-cell adhesion connections thus appears to have been already in place at the origin of the metazoans.

We can speculate on how Grh may have promoted metazoan multicellularity in light of other components we know to have been present at the metazoan ground state by referring to experiments on extant organisms. In the mouse, the Grh gene Grhl2 is crucial for the expression of the classical E-cadherin as well as claudin4, a junctional protein with homologs in sponges (Werth et al., 2010; Riesgo et al., 2014). E-cadherin promoter activity depends on an intronic enhancer, which binds Grhl2, and the absence of the TF leads to a specific decrease in acetylation and other activating histone modifications (Werth et al., 2010). In addition, Grhl2 has a nearly unique suppressive activity on gene activation by p300, exerted at the histone acetylase domain of the master coregulator. It performs this role in the negative regulation of several metalloproteinases, thereby inhibiting EMT and cell detachment events that lead to tubulogenesis (Pifer et al., 2016). Grainyhead TFs therefore have synergistic, reciprocal effects on promoting cohesion of cell clusters. They may have acted constitutively during early metazoan evolution, only to have become subject to downregulation in later-emerging complex forms in which EMT is a mode of development.

As noted, Grh can reduce expression of certain genes, but it can simultaneously increase expression of others. It shares this property with just a minority of TFs, referred to as “pioneer” regulators, which act at upper levels of enhancer hierarchies to prime target regions for the binding of activating or inhibitory cofactors (Jacobs et al., 2018; Mayran and Drouin, 2018; Gehrke et al., 2019). On this basis, it is possible that the repurposing of Grh was an originating step not only in metazoan multicellularity but also in metazoan developmental gene regulation. Once constituted with components inherited from unicellular ancestors (e.g., p300/CBP transactivating hubs, Grh factors, β-catenin, PcGII proteins) or emerging at the unicellular holozoan-metazoan interface (e.g., enhancers; classical cadherins and their cytoskeletal adaptors, including PcGI proteins), a new category of organism, a “proto-metazoan” could proceed to create a multiplicity of differentiated cell types.

### Cell Differentiation as the Amplification of Inherent Cell Functions

Opisthokont progenitors and ancestral holozoans likely exhibited alternative cell phenotypes and states. While some of these may have been antecedents of differentiated cell types in metazoans (Brunet and King, 2017), there are more than 250 cell types that characterize complex animals such as the vertebrates, and they all could not have arisen from ancestral ones. Furthermore, while GRNs for specific cell types (e.g., neurons, skeletal myoblasts) exhibit a fair degree of conservation across animal phyla (Arendt et al., 2016), regulatory mechanisms for the “same” cell types, and even for homologous genes show great variability across, phyla, species, and even in individuals within a species, often making assignment of similarly acting or appearing cell types as homologs ambiguous (Halfon, 2017). It has been suggested, for example, that neurons of ctenophores and cnidarians were independently evolved (Moroz and Kohn, 2016), although this remains controversial. As mentioned above, the mesoderm in triploblastic phyla might be polyphyletic (Burton, 2008), and within the deuterostomes, skeletogenesis is regulated by different TFs in chordates and echinoderms (Rafiq et al., 2012; Burke, 2018; Taylor and Heyland, 2018).

Cell-type homologies based on descent-with-modification exist because early-radiating members of a phylogenetic lineage can transmit their cell-type regulatory modes to their descendants. However, several phenomena, partly overlapping, contribute to “developmental system drift” that can disconnect cell types from their originating GRNs (True and Haag, 2001; Halfon, 2017). These include, as mentioned above, variable enhancer use across species, the fact that enhancers and promoters do not have entirely disjoint roles and can substitute for one another, the fact that developmental TFs have high concentrations of IDP domains and consequently operate at different cis-acting elements in different contexts, including different stages in the establishment of a cell-type lineage (Niklas et al., 2015; Masoudi et al., 2018), and the fact that a TF can act in entirely opposite fashions depending on the context (Murgan et al., 2015; Lukoseviciute et al., 2018).

Cell differentiation proceeds according to relatively routinized programs in present-day metazoans, but it has likely been less programmed earlier in evolution. The pan-metazoan secreted molecule Wnt, acting at the cell surface, typically initiates gene expression changes during early animal development utilizing a conserved (“canonical”) signal transduction pathway. However, the key transcriptional regulatory component of this pathway, β-catenin, which resides at cadherin-cytoskeletal linkages at the inner surface at the cell membrane until it is mobilized for this function, can also be activated at other pathway nodes by environmental influences, such as mechanical stress (Zhang et al., 2016) and hyperoxia (Popova et al., 2012). The most common co-transcriptional activators of β-catenin, the TCF/LEF family of transcription factors, can also be independently modulated, with numerous identified regulators of its transcription including the Wnt pathway itself (Cadigan and Waterman, 2012). Further, both β-catenin and TCF are highly promiscuous regarding the TFs they partner with, due to the intrinsically disordered (protein) TADs at the N- and C-terminal regions of β-catenin (Zhao and Xue, 2016). Moreover, β-catenin can also function independently of TCF/LEF (Doumpas et al., 2019). In ctenophores, deployment of β-catenin and TCF/LEF appears to entirely circumvent Wnt during early development (Pang et al., 2010).

Single-cell transcriptomic analysis of developmental systems shows a remarkably variable set of regulatory modes. In early *Drosophila* embryogenesis, for example, where the complex interaction of about 1,000 enhancers defines the activity of a few segmentation genes, some genes express across the field of cells in random bursts, others in broad, progressively refined patterns, and still others in sharp synchrony, to eventually achieve precise and reliable levels of expression (Vera et al., 2016; Bothma et al., 2018). Spatiotemporal precision, as discussed in the following section, is probably imposed at a different level of organization from that on which cell type identity is determined.

A highly plastic but ultimately globally controllable transcriptional regulation apparatus that distinguished among housekeeping, developmental, and specialty genes thus arose coordinately with Metazoa and was in operation in basal metazoans like sponges (though apparently not fully in placozoans). The subsequent appearance of 200 or so cell types was the result of recruitment of this metazoan ground state system of gene control for the enhancement and elaboration of preexisting (and thus inherent) unicellular physiological functions. Whether one or more of the cells in a multicellular entity performed any of these intrinsic functions, e.g., motility, extracellular matrix production, light or nociception, oxygen capture, fuel storage, detoxification, and so forth, may or may not have contributed to the survival of the novel differentiated form. This was decided by natural selection (based on possibilities afforded by the new feature) or construction and occupation of previously unexploited niches.

“Proto-cell types,” providing, for example, evolutionary starting points for the evolution of muscle, neurons, connective tissue, blood, glands, and liver, need not have originated *via* germline encoding (in this scenario, the germline itself may have arisen by this same “plasticity first” process), or in a reproducible spatiotemporal or even quantitatively regulated fashion. That is, the novel cellular phenotypes could have appeared in a cell mass randomly or reversibly. More programmed modes of appearance could have followed through the self-organizational properties of a critical mass cells (see the following section) and by genetic assimilation, once a germline was in place (Newman, 2011).

The most enigmatic ingredient in this scenario for the transition from the metazoan ground state to complex animals with scores or hundreds of cell types was the appearance of LDTFs and related TFs. These initiate the expression of suites of genes that start an embryo or organ primordium on a progressive developmental trajectory leading, in a hierarchical cascade, to one or more terminally differentiated states (Obier and Bonifer, 2016). The LDTFs include such proteins as MyoD, Nkx3-1, neurogenin, Sox9, and PPAR-γ, which specify (with accessory factors), skeletal muscle, heart muscle, neurons, cartilage, and fat, respectively (reviewed in Newman et al., 2009). Included among these are pioneer TFs, like Grh. Some LDTFs act in a mutually antagonistic fashion ensuring, in concert with silencing effectors like PcGI proteins, that mixed-identity cell types are not produced (Sunadome et al., 2014). Although proteins related to certain LDTFs are present in the genomes of unicellular holozoans or yeast, most are novelties of Metazoa (Grau-Bové et al., 2017). They are therefore orphan genes like many of those involved in the morphogenetic innovations described above in “Inherent Forms in Animal Origination and Evolution.” Although there are speculations on the origins of such genes (Bornberg-Bauer et al., 2015; Neme and Tautz, 2016; Schmitz et al., 2018; Werner et al., 2018), there are no generally accepted accounts for the connection between their appearance and their roles in the recruitment of ancestral cellular functions for cell differentiation.

Finally, it is important to recognize that while cell types in mature metazoans are typically functionally stable, wound repair and regeneration require cell type plasticity. The mechanisms described above are reversible under certain conditions, consistent with this need (Wenger et al., 2016; Gehrke et al., 2019), a phenomenon also manifested in the experimental production of stem cells (Sardina et al., 2018).

## Pattern Formation: Inherency in the Integration of Form and Function

We have seen how the innovations in form and function that accompanied the transition from unicellular holozoans to the metazoans were fundamentally expressions of latent, inherent properties of this new category of organism. To reiterate, hollow, multilayered, elongated, appendage-bearing diploblastic and triploblastic forms were direct consequences of the mobilization of mesoscale physical forces and effects in liquid and liquid-crystalline cell aggregates. The existence of such multicellular aggregates and the presence within them of cell types specialized to perform exaggerated versions of ancestral unicellular functions were direct consequences of the new type of enhancer- and silencer-based gene regulatory systems that emerged simultaneously with the metazoans. While inherent to metazoans, morphogenesis and differentiation were not present in their unicellular ancestors. New genes proteins, and regulatory motifs (classical cadherins, Wnt, enhancers), several of which with no known predecessors, were needed to create these inherencies.

The capacity to arrange differentiated cell types in reproducible geometries to produce functionally adequate organs and appendages, that is, cellular *pattern formation*, is still another category of phenomena with inherent and therefore predictable outcomes. Once the basic ingredients, i.e., pan-metazoan toolkit genes and their products, and the physics of permeable, active, and excitable media to which animal tissues are intrinsically susceptible, were in place, many of the shared features of animal functional anatomy emerged with relative facility.

### Morphogens and Lateral Inhibition

The simplest way cells in a tissue mass can be induced to differentiate in a spatially dependent fashion is by *morphogens*, secreted diffusible proteins. Wnt, as noted above, is the most fundamental and ubiquitous of these in the metazoans, but there are more than a dozen widely shared ones, including those of the Hedgehog family, such as Shh and Ihh, Bmps, and Fgfs (reviewed in Newman and Bhat, 2009). Any molecule of this type produced in a cluster of ancestral cells could easily have become nonuniformly distributed by leakage from the surface of the mass, or possibly due to poor nutrient supply in the interior leading to lower expression of its gene. Since such conditions would automatically have been reproduced each time a cell cluster was reconstituted, the morphogen and its physically determined spatial arrangement, combined with its concentration-dependent effects in eliciting changes in gene expression or other aspects of cell phenotype (Loh et al., 2016), may have constituted a primitive developmental program. Subsequent natural selection would have reinforced the accuracy and reliability of such pattern-forming modalities if the resulting organisms proved to be survivable. Eventually the transport of morphogens has come to be not only due to diffusion but to active processes mediated by cells along the distribution routes (Sagner and Briscoe, 2017). In the perspective of the present analysis, however, a key feature of these mechanisms is their origins in inherent properties of collectives of metazoan cells.

In addition to morphogen-based global patterning, the phenomenon of *lateral inhibition* enforces a choice between alternative cell fates at the local level. This is accomplished by early differentiating cells signaling to cells adjacent to them to take on a fate different from them and is typically mediated by the Notch signal transduction pathway, which is found in all metazoan groups with the exception of Placozoa (reviewed in Newman, 2016b). The activity of this pathway depends on the binding of a member of the Notch family of cell surface receptors, with an integral membrane protein ligand from among the Delta, Serrate (or Jagged), and Lag-2 (DSL) class of proteins (Ehebauer et al., 2006). This interaction results in an intracellular portion of Notch translocating to the nucleus where, as a transcriptional co-regulator of dual-action transcription factors of the CSL (CBF1, Su(H), and Lag-1) class (Lai, 2002), changes them from repressors to activators of key target genes. Since Notch’s effects on cell phenotype depend on which of the dual-action factors are present in a given cell type, the pathway does not determine the cell’s fate, but rather causes it to choose a potential fate different from the one it would have it assumed without the juxtacrine (i.e., direct cell-to-cell) signal.

The Notch pathway, like many of the metazoan morphogenesis and differentiation mediators discussed above, is unique to this group, although some components or portions of them can be recognized in earlier emerging forms. Protein modules of Notch receptors are present in the choanoflagellate holozoan *Monosiga brevicollis* (King et al., 2008) and a Notch receptor, ligands, and an intracellular mediator are present in the filasterean holozoan *Ministeria vibrans* (Shalchian-Tabrizi et al., 2008). Studies on CSL homologs in yeast indicate that their role in determining alternative cell states is evolutionary deeper than the holozoans (Prevorovsky et al., 2015).

The physical behaviors of the biogeneric materials discussed earlier in this article – rounding up, phase separation, lumen formation, elongation, wetting, condensation – notwithstanding their dependence on living cells – are analogs of those that result from passive arrangements and rearrangements of subunits in nonliving liquids. Here, however, with the effects of morphogen induction and lateral inhibition in eliciting alternative cell states, we see analogs to chemical transformations. Like chemistry, these are active (i.e., energy consuming) rather than passive processes, in which one kind of entity (a molecule or a cell type) is changed into another. The simplest of these effects is unidirectional, a morphogen choosing the fate of a cell from among the organism’s repertoire of types, or a cell imposing a choice on a second cell. When there are reciprocal influences – positive or negative feedbacks, or synchronization – among the components, the systems can become not merely active, but excitable (i.e., energy storing and releasing; self-organizing (Süel et al., 2006; Sinha and Sridhar, 2015)], and more complex patterns can emerge.

### Oscillations and Morphogenetic Fields

Lateral inhibition mediated by the Notch pathway is well suited to generating fine-grained salt-and-pepper-like patterns in which adjacent cells are of different types. Organogenesis in complex animals, however, depends on large-scale coordination of patterns of cell differentiation in which patches and domains of cells of a given type—tissues—associate in specific geometric arrangements with other such domains in the architecture of body layers, appendages, and primordia arising from the morphogenetic processes described above. Morphogen gradients, mentioned above, can mediate long-range control of differentiation, but in order to effect accurate pattern formation, the cells in the targeted regions need to be in similar states of receptivity. This might appear to be a property that is not intrinsic to cells in developing tissue primordia and must be a product of adaptive selection. But, it can in fact arise spontaneously from mechanisms and ingredients that have evolved for other reasons.

A main target gene of the CSL transcription factors at the end of the Notch signal transduction pathway is Hes1 (called hairy1 in Drosophila). Hes1, itself a transcriptional regulator, has the unusual property of regulating its own gene expression through a negative feedback mechanism. Due to this feedback control, the level of Hes1 protein in expressing cells will tend to undergo temporal oscillation (Lewis, 2003). Then, through a well-described physical phenomenon of synchronization of weakly coupled oscillators (Garcia-Ojalvo et al., 2004), the cells in a spatially extended domain of tissue will find themselves in identical though periodically changing states of Hes1 concentration and of any other biochemical factor Hes1 controls. This poises them for concerted response to an external regulatory factor such as a morphogen. The Notch pathway and its downstream effectors, therefore, not only enable cells to directly influence the fates of their nearest neighbors through lateral inhibition, but facilitate the emergence of a “morphogenetic field” (Gilbert et al., 1996; Bhat et al., 2019). This phenomenon was described by early embryologists as “a system of order such that the positions taken up by unstable entities in one part of the system bear a definite relation to the position taken up by other unstable entities in other parts of the system” (Needham, 1937, p. 71).

The cell state-coordinating effect of synchronized oscillations of Hes1 expression or potentially other factors with similar feedback dynamics has a propensity (not invariably realized) of producing spatially periodic tissue structures. Hes1 oscillations are synchronized, for example, in the lateral plate mesoderm of vertebrate embryos, so that cells in any band of tissue along the cranial-caudal axis have approximately the same (periodically varying) Hes1 concentration (Özbudak and Lewis, 2008). The cells will aggregate, due to upregulation of adhesive molecules, when a certain Hes1 level is reached, but only when they are not inhibited from doing so by a graded suppressive activity, a morphogen, that emanates from the tail tip. As the embryo elongates, successive bands of tissue become distant enough from the caudal end to escape from the morphogen’s effect. When the Hes1 oscillation brings the factor’s concentration into the adhesion-inducing range in the newly competent cells (uniformly, because of synchrony), a mesenchymal-epithelial transformation takes place, and discrete blocks of tissue, “somites,” form to either side of the central axis (Hubaud and Pourquie, 2014). This process continues as long as the embryo elongates, generating a species-characteristic number of somites, which depends on both the rate of elongation and the period of the oscillator. While the somites give rise to various spatially periodic (the vertebrae and the ribs) and nonperiodic (the dermis of the back skin, and the muscles of the back, body wall, and limbs) structures, the manner in which they are generated suggests that the emergence of tandem tissue blocks is the side effect of various inherent processes (feedback regulation, morphogen action) that only incidentally, under certain mutually tuned conditions, lead to a repetitive spatial pattern.

In a similar fashion, Hes1 oscillations across the digital plate in the embryonic avian limb become synchronized during skeletal pattern formation. This enables spatial cues for skeletogenesis (see below) to act in a coordinate fashion over larger distances across the cellular field than the natural wavelength of their generating mechanisms (Bhat et al., 2019).

### Reaction-Diffusion Systems

Morphogens do not only function in the form of simple gradients to induce patterns of cell differentiation. By interacting with biosynthetic processes (including gene expression) in target cells and tissues, they can assume more elaborate geometries, which lead to more complex cell arrangements. Alan Turing, in a paper titled “The chemical basis of morphogenesis,” showed that a balance of positive and negative feedbacks in an open chemical system, when coupled with differences in the rates of diffusion of the key reactive molecules, could lead (contrary to the expectation that diffusion evens things out) to stable, nonuniform concentration patterns (Turing, 1952). These are referred to as “reaction-diffusion” mechanisms, and in developmental biology, “Turing-type” systems, but when living tissues are concerned neither “reaction” (actually cell-based production of, and response to, molecular factors) nor “diffusion” (or more generally, transport of released molecules through and between cells, with the latter’s active participation), are typically like those seen in purely chemical systems. Moreover, the core processes may differ substantially from Turing’s, lacking some of the original features, e.g., the difference in diffusivities of the chemical species involved, and containing others, e.g., chemotaxis and advection (see, for example, Glimm et al., 2014; Madzvamuse et al., 2015; Nesterenko et al., 2017).

Turing-type mechanisms pattern cell types in a variety of embryonic systems, including spatially nonperiodic ones such as the generation of left-right asymmetry (Muller et al., 2012) and the formation of individual tooth cusps (Salazar-Ciudad, 2012). But, it is in the production of repetitive or quasi-repetitive structures such as hairs and feather germs (Glover et al., 2017; Painter et al., 2018), and pigment patterns in skin (Haupaix et al., 2018), that this category of self-organizing process has found its broadest applications. The paired appendages – fins or limbs – of the jawed vertebrates, for example, are characterized by arrangements of cartilaginous and/or bony elements that appear to have arisen evolutionarily by and continue, to some extent, to be mediated by such mechanisms. In cartilaginous fishes such as sharks, skates, and rays, the fin skeleton consists of one or more cartilage rods or plates to which are appended as many as several dozen parallel, jointed cartilage rods. In ray-finned fishes (carp, zebrafish, and so forth), the endoskeleton is made of plates, rods, and nodules of cartilage and bone. Finally, in the lobe-finned fish like the coelacanth, and tetrapods, such as amphibians, birds, and mammals, the cartilaginous or bony skeleton is comprised of increasing numbers of parallel elements along the proximodistal axis originating at the body wall (Newman et al., 2018).

Evidence from experimental embryology and molecular genetics, phylogenomics, and mathematical and computational simulations has provided plausible scenarios for transitions, often abrupt, between these patterns over the course of evolution of the paired appendages resulting from subtle changes in transcription factor-cis-regulatory element binding within conserved GRNs, and protein-protein interactions at the cell surface. In these cases, and in others which have been explored in detail, active and excitable mechanisms of cellular differentiation and pattern formation, in coordination with more passive morphogenetic mechanisms of the liquid- and liquid crystalline-tissue states, each utilizing genes, molecules, and regulatory motifs particular to metazoan life, lead to the formation of recognizably animal-type tissues and organs with the capacity to perform functions carried over from ancestral cells.

## Conclusion: Predictability of Animal Organization and Integration

This review has described how three main properties of animal bodies and organs, their forms, their differentiated cell types, and the patterned arrangement of cells of various types, are determined by inherent properties of aggregates of metazoan cells. These inherent properties are not all of the same type. The only such properties that were actually latent in directly ancestral unicellular organisms are those manifested as the specialized functions of cell types. As indicated above, unicellular holozoans are variously capable of adhering to one another, absorbing molecules and ingesting particulate nutrients, exhibiting contractility and electrical excitability, surrounding themselves with semisolid or solid matrices, binding oxygen, neutralizing toxins, and excreting waste products. All of these functions appear in exaggerated form in one or more of the 200–300 animal cell types. Functions inherent to the ancestors of other multicellular lineages, such as the capture of light and the storage of its energy in sugars in vascular plants, appear in differentiated cells of those organisms, but not the animals.

As noted above, the recruitment of inherent cell functions for cell differentiation sometimes employed transcription factors which controlled the relevant function-related genes in the unicellular ancestor. Most often, however, the differentiation and other developmental TFs are not found outside the metazoans (Grau-Bové et al., 2017). In all cases, however, the TFs involved in cell differentiation became incorporated into hierarchies, based on the novel and unique gene regulatory apparatus of Metazoa that distinguished their roles in establishing cell types from those in maintaining them, and set both of these apart from regulation of gene expression in nonspecialized “housekeeping” activities.

The relevant organizational properties for morphogenesis and pattern formation are those characteristic of mesoscopic materials (e.g., liquid- and liquid-crystalline tissues) or extended excitable media (e.g., “diffusion” and “reaction,” synchronization of oscillations). While the respective physical effects are therefore generic to such materials, the concept of inherency was only applicable once the materials came into existence as multicellular masses. It is notable that gene innovations that are specific, or now uniquely confined, to Metazoa were required for such materials to be constituted and thus for the inherent properties of their physical state to be realized.

The fact that body and organ forms, cell and tissue functions, and cellular patterns are inherent to animal systems has major consequences for understanding evolution (Newman, 2017). (See Webster and Goodwin, 1996 and Amundson, 2005 for accounts of the structuralist tradition in evolutionary theory, in which inherency is an important theme.) Inherency clearly does much of the work attributed in the standard model to trial-and-error-based natural selection. But inherency of form and function does not mean simultaneous emergence of all possibilities. The main animal types do not appear in the fossil record prior to strata deposited tens of millions of years after the first signs of metazoan-type organisms, more than 600 million years ago. With the exception of placozoans and ctenophores, which, respectively, implement the developmental gene-regulatory and liquid-tissue constituting activities of enhancers and classical cadherins differently, the capability to mobilize pre-existing holozoan cell functions in morphologically varied, spatially patterned assemblages was present from the start and carried forward in all subsequent evolution of this group. Additional complexity was achieved mainly by the addition of new genes specifying ECM components.

Finally, the remarkable phenomenon of electrical integration of multicellular patterns must be mentioned. Although thus far not demonstrated to be a primary form-generating or patterning mechanism like differential adhesion, morphogen gradients, gene expression oscillation, and reaction-diffusion coupling, bioelectric establishment of voltage gradients across tissues serves to reinforce developmental patterns and restore them if perturbed during embryogenesis or wounding (McLaughlin and Levin, 2018; Pietak and Levin, 2018). Voltage-gated channels with single-cell functions are inferred to have existed in unicellular holozoan ancestors of the animals (Cai, 2012), but their developmental role in the multicellular context is entirely different from any function they evolved to perform in individual cells. Along with canalizing evolution that serves to stabilize the phenotype of an evolutionary lineage against mutation and developmental noise once it has become ecologically established, the dynamically responsive encoding of morphology by bioelectricity is a key factor in organismal autonomy (Arnellos et al., 2014; Moreno and Mossio, 2015).

Major steps in animal evolution not only occurred by such repurposing of ancestral genes (as we also saw in the case of the grainyhead transcription factor), but also involved in the appearance, often without precedent, of novel genes whose products set into play forces and effects, which though having predictable outcomes, were unavailable up to the points at which they appeared (Table 1).

Natural selection can act as a sieve for organisms with novel combinations of inherent characters, or characters that become inherent to a lineage after genetic augmentation, but it is not responsible for producing those traits. The characters’ origination, whatever the source of the associated genes, can often only be understood on the basis of physico-genetic effects specific to the multicellular context. Characters produced in this fashion are constrained in their nature and may therefore appear abruptly (McGhee, 2011). If they enable organisms to survive in new ways in existing ecological niches, or to occupy new niches (Laland et al., 2017), their roles in enhancing fitness will be “after-the-fact” (Gould and Lewontin, 1979; Müller, 1990; West-Eberhard, 2003) and thus do not require elaborate or farfetched adaptationist narratives to account for their existence.

## Author Contributions

The author confirms being the sole contributor of this work and has approved it for publication.

### Conflict of Interest Statement

The author declares that the research was conducted in the absence of any commercial or financial relationships that could be construed as a potential conflict of interest.

## Footnotes

^1^Glass sponges, of the poriferan class Hexactinellida, have syncytial tissues. Although they contain and utilize the “canonical” Wnt pathway (see infra), they lack the extracellularly acting Wnt morphogen itself (Windsor Reid et al., 2018).

^2^There are differences in the literature on what constitutes a “true” epithelium. Here, since my focus is on physical distinctions among tissue types and their role in phyletic transitions, I reserve this term for planar tissues underlain by a basal lamina, which are present in all eumetazoans and in the homoscleromorph sponges. Others use the term more broadly for any cell layer with junctional complexes that permit it to serve a barrier function, a feature found in all sponge groups (Leys and Riesgo, 2012).

^3^The placozoan *Trichoplax* has a peroxidasin gene but lacks a basal lamina (Fidler et al., 2017), possibly due to the absence of the requisite crosslinkable amino acid residues in its type IV collagen molecules (Fidler et al., 2014).

^4^The tissue layers of most sponge groups exhibit morphological plasticity that allows them to assume both epithelium- and mesenchyme-like configurations, though these are transient and interconvertible, and thus physically different from the germ layers of triploblasts. Within the quasi-mesenchyme consisting of the extracellular matrix (mesohyl) and its resident cells, however, some sponges species form a spicule-containing endoskeleton (Leys and Hill, 2012).
