# Corrigendum: Inherency of Form and Function in Animal Development and Evolution

**DOI:** 10.3389/fphys.2019.01433

**Published:** 2019-12-03

**Authors:** Stuart A. Newman

**Affiliations:** Department of Cell Biology and Anatomy, New York Medical College, Valhalla, NY, United States

**Keywords:** biogeneric, morphogenesis, pattern formation, cell differentiation, self-organization, phase transition, reaction-diffusion

In the original article, there was a mistake in Table 1 as published. Because of an editing error, the lines for “Apicobasal cell polarization” and “Nonliquid cellular assemblages *via* matrices” were transposed. The corrected Table 1 appears below.

**Table 1:** Novel inherent properties in animal development and evolution.

**Property**	**Gene or molecular motif**	**Character**
1. Properties dependent on novel genes or regulatory motifs coincident with emergence of Metazoa Liquid-tissue state Regulated cell polarity Capacity to exaggerate intrinsic cell functions Morphogen gradients	Classical cadherins Wnt Enhancers; PcGI proteins Hedgehog, BMPs	Multicellularity; layering Lumens; elongated tissues Differentiation Simple cell patterns
2. Properties dependent on novel genes acquired after metazoan origins Liquid-crystalline-tissue state Wettable substrata (basal lamina) Lateral inhibition; oscillation of gene expression Multiple alternative cell types	Vang/Stbm Peroxidasin Notch, Hes1 MyoD, PPARy, SMAD	Tissue elongation Appendages, glands Complex cell patterns Complex tissues, organs
3. Properties dependent on ancestral genes repurposed into DPMs in the multicellular context Cell-cell cohesion in liquid tissues Apicobasal cell polarization Nonliquid cellular assemblages *via* matrices Cell-cell electrical coupling	Grainyhead β-catenin Collagen IV Voltage-gated channels	E-M transformation Epithelia and lumens Mesenchymal tissues Bioelectrical integration

*Each list is nonexhaustive but contains the most important examples of its respective category*.

The author apologizes for this error and states that this does not change the scientific conclusions of the article in any way. The original article has been updated.

